# Prevalence of Overweight, Obesity, and Comorbid Conditions Among U.S. and Kentucky Adults, 2000–2002

**Published:** 2004-12-15

**Authors:** Todd M Jenkins

**Affiliations:** University of Kentucky Prevention Research Center, Lexington, Ky

## Abstract

**Introduction:**

Obesity rates for adults in Kentucky are regularly among the highest in the nation. Since 1991, adult obesity in Kentucky and the United States has nearly doubled. This trend is of great concern because excess weight has been associated with several chronic diseases and conditions. This paper reports on the prevalence of overweight and obesity among adults in Kentucky between 2000 and 2002. The estimates produced by this study will provide baseline figures for developing Kentucky's statewide obesity action plan.

**Methods:**

A secondary data analysis was performed using the Centers for Disease Control and Prevention's Behavioral Risk Factor Surveillance System. Prevalence estimates and odds ratios were calculated for the United States and Kentucky.

**Results:**

In Kentucky, 24.2% of adults were obese, compared with 21.9% nationally (*P* < .001). There were also significantly more overweight adults in Kentucky than there were nationwide (*P* < .001). Logistic regression showed that overweight and obese adults were more likely to report various comorbid conditions.

**Conclusion:**

Overweight and obesity estimates in Kentucky were significantly higher than nationwide figures. However, overweight/obese adults in Kentucky were no more likely than their U.S. counterparts to report selected comorbid conditions.

## Introduction

The obesity epidemic in the United States worsens with each passing year. From 1991 to 2002, the prevalence of obesity has increased more than 80%, representing an estimated 43 million adults in 2002 (1). In 1991, no state in the nation had an obesity prevalence at or above 20%, but by 2002 there were 39 states with this characteristic (2). The severity of this epidemic has been highlighted in *Healthy People 2010*, where overweight and obesity have been grouped as leading health indicators for the United States (3). In terms of mortality, an estimated 280,000 to 325,000 adults in the United States die each year from causes related to obesity (4). More importantly, excess weight has been positively correlated with years of life lost (5).

In addition to mortality, substantial morbidity is associated with obesity. For example, in 2000, the total cost of obesity in the United States was estimated to be $117 billion ($61 billion in direct medical costs, $56 billion in indirect costs) (3,6). An estimated 9.1% of annual medical spending in the United States is attributed to overweight and obesity — a figure that rivals medical costs attributable to cigarette smoking (7). Overweight and obesity have been associated with several chronic diseases and conditions, including cardiovascular disease, type 2 diabetes, hypertension, stroke, arthritis, high serum cholesterol, and some cancers (8-10). This is of great concern in Kentucky because the prevalence rates for overweight and obesity continue to increase and are regularly among the highest in the nation. All told, obesity substantially increases morbidity and impairs quality of life (11). Kentucky is developing a statewide action plan to address this public health issue. Estimates produced from this analysis will serve as baseline figures for the action plan.

## Methods

A secondary data analysis was performed using data from the Behavioral Risk Factor Surveillance System (BRFSS), 2000–2002 (12,13). Conducted by the Centers for Disease Control and Prevention (CDC), the BRFSS is an annual population-based, random-digit-dialed telephone survey of the noninstitutionalized U.S. civilian population aged 18 or older. This ongoing surveillance system measures health behaviors and preventive practices related to several leading causes of death (12,13). Kentucky data were obtained from the Kentucky BRFSS Program (KY BRFSS) (14). Data from 21,016 adults in Kentucky were collected during this period. U.S. data were retrieved from the CDC's public-use BRFSS datasets (15). Data from 642,924 adults across the nation were collected during 2000–2002. The national dataset included data from Guam, Puerto Rico, and the Virgin Islands, but these areas were excluded from this analysis.

Overweight and obesity classifications used in the analysis were derived from Body Mass Index (BMI) and were consistent with the definitions set forth by the World Health Organization (WHO) and the National Heart, Lung, and Blood Institute (underweight: BMI<18.5; normal weight: BMI = 18.5–24.9; overweight: BMI = 25.0–29.9; obesity-class 1: BMI = 30.0–34.9; obesity-class 2: BMI = 35.0–39.9; obesity-class 3: BMI ⩾40.0) (9,16). BMI (calculated as weight in kilograms divided by the square of height in meters) was calculated using the following questions: 1) "About how much do you weigh without shoes?" and 2) "About how tall are you without shoes?" (17). Respondents with missing or unknown height or weight data were excluded from the analysis. Women who reported they were pregnant at the time of the interview were also excluded from the analysis. After all exclusions, a total of 590,120 respondents for the United States and 19,722 respondents from Kentucky were included in the analysis.

Comorbid conditions were measured using the following questions (17):

**Diabetes**. Have you ever been told by a doctor that you have diabetes? (2000–2002)

**Asthma.** Did a doctor ever tell you that you have asthma? (2000) Have you ever been told by a doctor, nurse, or other health professional that you have asthma? (2001–2002)

**Arthritis. **Have you ever been told by a doctor that you have arthritis? (2000–2001) Have you ever been told by a doctor or other health professional that you have some form of arthritis, rheumatoid arthritis, gout, lupus, or fibromyalgia? (2002)

**High blood pressure. **Have you ever been told by a doctor, nurse, or other health professional that you have high blood pressure? (2001)

**High cholesterol. **Have you ever been told by a doctor, nurse, or other health professional that your blood cholesterol is high? (2001)

**Health status. **Would you say that in general your health is: Excellent, Very Good, Good, Fair, or Poor? (2000–2002).

Women reporting gestational diabetes were coded as having diabetes. Questions assessing blood pressure and cholesterol are asked in alternating years (rotating core questions) and were not selected as modules in most states (including Kentucky) in 2000 or 2002; thus, data from these questions were analyzed for 2001 only.

SAS version 8.2 (SAS Institute Inc, Cary, NC) and SAS-Callable SUDAAN version 8.0.1 (Research Triangle Institute, Research Triangle Park, NC) were used to perform the data analysis and to account for the complex sampling design (18,19). PROC DESCRIPT was used to calculate age-adjusted prevalence estimates and their corresponding standard errors. Prevalence estimates of obesity, overweight, and comorbid conditions were age-adjusted to the 2002 BRFSS. However, figures representing total number of adults were derived from crude estimates. Multivariate logistic regression was performed using PROC RLOGIST to assess associations between BMI and comorbid conditions while controlling for age, race, sex, education, and smoking status. All reported data are weighted, correcting for variation in selection probability and demographic imbalances (20).

## Results

During the years 2000–2002, 24.2% of adults (683,000) in Kentucky were obese (BMI ⩾30.0), compared with 21.9% (42 million) in the United States (*P* < .001) (Table 1). Both men (24.6%) and women (23.8%) in Kentucky had significantly higher levels of obesity compared with men and women nationally (21.9% [*P* < .001] for men and 21.7% [*P* < .001] for women). Among race/ethnicity groups, only non-Hispanic whites in Kentucky had a significantly higher obesity estimate compared with the United States (*P* < .001).

There were also more overweight (BMI = 25.0–29.9) adults in Kentucky than nationwide. During 2000–2002, 62.8% of adults in Kentucky (1.76 million adults) were overweight, compared with 59.7% (115 million adults) nationally (*P* < .001) (Table 2). As seen with obesity, the prevalence of overweight among men (70.7%) and women (54.8%) in Kentucky was also significantly higher than among their counterparts nationally (67.9% [*P* < .001] of men and 51.3% [*P* < .001] of women). Estimates for overweight were also higher among non-Hispanic whites in Kentucky (62.3%) compared with the United States (57.8% [*P* < .001]). Rates for non-Hispanic blacks in Kentucky and in the United States were not significantly different, but they were significantly higher than for non-Hispanic whites within both regions. By age group, estimates peaked at ages 50–59 for both Kentucky (70.0%) and the United States (67.6%).

Table 3 lists the prevalence of comorbid conditions by BMI category among Kentucky and U.S. adults. As expected, the prevalence of each condition increased with BMI. The largest increases in prevalence were observed with diabetes and fair or poor health status. For comparisons between Kentucky and the United States, differences were most pronounced for arthritis and fair or poor health status. For every BMI category, the prevalence of adults in Kentucky with arthritis was greater than adults nationally. The prevalence of adults in Kentucky reporting fair or poor health status was higher in the United States for all but the highest BMI category, obesity-class 3.

Multivariate logistic regression analysis indicated significant associations for overweight and obesity with each comorbid condition (Table 4). Overweight and obese adults in Kentucky were more likely than those of normal weight to have diabetes, asthma, arthritis, high blood pressure, high cholesterol, and fair or poor health status. As expected, results were strongest for those with obesity-class 3. Using a normal BMI as the reference, the odds of Kentucky respondents with obesity-class 3 were nine times higher (Prevalence Odds Ratio [POR] 9.10) to report diabetes, four times higher (POR 4.26) to report arthritis, more than six times higher (POR 6.83) to report high blood pressure, and more than four times higher (POR 4.59) to report a fair or poor health status. However, none of the results listed in Table 4 for Kentucky was significantly different from U.S. estimates.

## Discussion

Since 1991, the prevalence of obesity among adults in the United States and Kentucky has doubled (1,21). When combined with overweight, more than 60% of adults throughout the United States and Kentucky are classified as overweight/obese (BMI ⩾25). Among the fifty states and the District of Columbia in 2002, the obesity rate in Kentucky was the 13^th^ highest (24.4%; 95% confidence interval [CI], 22.8–26.1), 8.5% higher than the U.S. rate (22.5%; 95% CI, 22.2–22.8). For overweight, Kentucky had the sixth highest estimate (63.6%; 95% CI, 61.8–65.4), 5.3% greater than the U.S. rate (60.4%; 95% CI, 60.0–60.7).

Results by age indicate that younger adults in Kentucky (aged 18–29, 30–39, 40–49) had significantly higher obesity estimates than younger adults in the United States. Data for overweight were similar, with estimates for adults up to the age of 60 significantly higher in Kentucky. Comparisons between the United States and Kentucky for youth (<18 years) were also similar. According to the 2001 Youth Risk Behavioral Surveillance System (YRBSS), 12.3% of high school students in Kentucky were overweight, and another 15.2% were at risk for becoming overweight, compared with 10.5% overweight and 13.6% at risk nationally (22). These data suggest that the prevalence of overweight and obesity is unlikely to change in Kentucky in the foreseeable future. Results from this analysis revealed that overweight and obesity are more prevalent in Kentucky, but those with excess weight were no more likely to have other comorbid conditions (e.g., diabetes, arthritis) in Kentucky than observed nationally. However, with its disproportionate share of overweight and obesity, Kentucky will face the costly task of treating and caring for a disproportionately greater number of its population beset with comorbid conditions related to excess weight for many years to come.

The results reported here are subject to several limitations. First, the survey design includes only those noninstitutionalized civilian adults who have a telephone. Therefore, results are generalizable only to this population. According to Census 2000, 2.4% of occupied housing units across the nation and 4.7% in Kentucky do not have telephone service (23). Individuals without telephones are more likely to have a low socioeconomic status, which is associated with obesity (24,25). Therefore, results in this analysis are likely to be underestimated. The use of self-reported height and weight represents another limitation. Respondents in self-reported surveys tend to overestimate their height, while overweight respondents tend to underestimate their weight (1). Compared with studies based on directly measured height and weight, such as the National Health and Nutrition Examination Survey (NHANES), obesity estimates from self-report tend to be lower (26). The prevalence of obesity from NHANES 1999–2000 was 30.5%, compared with 19.8% from the 2000 BRFSS (10,26). There are also drawbacks to using BMI as an indicator for overweight and obesity. BMI can overestimate body fat in persons who are very muscular and underestimate body fat in persons who have lost muscle mass, such as many elderly (10). However, estimates from these potentially misclassified groups likely had little overall impact on the analysis.

The impact of excess weight extends beyond the monetary costs and physical ailments associated with it. Other issues such as social stigma, discrimination, and poor body image all contribute to a lower quality of life for the overweight and obese compared with individuals of normal weight (3,27). If current trends continue, obesity will soon overtake smoking as the primary preventable cause of death (28). These results, in part, serve as baseline figures for Kentucky's initial obesity action plan. Future initiatives addressing diet and physical activity are anticipated to be derived from this plan.

## Figures and Tables

**Table 1:** Prevalence of Obesity (Body Mass Index ≥30) by Demographic Characteristic, Adults Aged ≥18, United States and Kentucky, 2000–2002 Behavioral Risk Factor Surveillance System^a^

**Characteristic**	**Kentucky**	**United States**
**Total**	24.2 (23.4-25.1)	21.9 (21.7-22.1)

**Sex**		

Male	24.6 (23.2-25.9)	21.9 (21.7-22.2)
Female	23.8 (22.6-24.9)	21.7 (21.5-22.0)

**Race/ethnicity**		

Non-Hispanic white	23.7 (22.8-24.6)	20.3 (20.1-20.5)
Non-Hispanic black	33.7 (29.2-38.2)	32.9 (32.2-33.6)
Non-Hispanic other	24.4 (17.2-31.5)	16.5 (15.7-17.4)
Hispanic	21.0 (14.2-27.7)	26.2 (25.1-27.2)

**Age (years)**		

18-29	18.4 (16.0-20.9)	14.3 (13.9-14.7)
30-39	26.9 (24.7-29.2)	21.4 (21.0-21.8)
40-49	28.2 (26.1-30.2)	24.6 (24.1-25.1)
50-59	27.0 (24.9-29.1)	26.9 (26.4-27.4)
60-69	25.2 (22.9-27.4)	25.2 (24.6-25.7)
70+	17.0 (15.2-18.9)	17.4 (16.9-17.8)

**Education**		

<High school	28.2 (26.1-30.3)	29.4 (28.6-30.1)
High school grad	25.1 (23.6-26.5)	24.3 (23.9-24.6)
Some college	25.4 (23.4-27.3)	22.3 (21.9-22.6)
College+	18.0 (16.1-19.8)	16.2 (15.9-16.5)

**Smoking status**		

Current	19.6 (18.2-21.1)	17.5 (17.1-17.8)
Former	28.6 (26.0-31.3)	24.0 (23.6-24.4)
Never	25.2 (23.9-26.5)	22.5 (22.2-22.7)

a All values represent percentages (95% confidence intervals). Age-adjusted to the 2002 Behavioral Risk Factor Surveillance System.

**Table 2:** Prevalence of Overweight (Body Mass Index = 25.0–29.9) by Demographic Characteristic, Adults Aged ⩾18, United States and Kentucky, 2000–2002 Behavioral Risk Factor Surveillance System^a^

**Characteristic**	**Kentucky**	**United States**
**Total**	62.8 (61.8-63.8)	59.7 (59.5-59.9)

**Sex**		

Male	70.7 (69.3-72.1)	67.9 (67.6-68.2)
Female	54.8 (53.5-56.1)	51.3 (51.0-51.6)

**Race/ethnicity**		

Non-Hispanic white	62.3 (61.3-63.3)	57.8 (57.5-58.0)
Non-Hispanic black	71.0 (66.6-75.5)	70.7 (70.0-71.4)
Non-Hispanic other	62.3 (53.4-71.2)	51.3 (50.0-52.6)
Hispanic	58.2 (49.7-66.8)	67.3 (66.3-68.3)

**Age (years)**		

18-29	50.7 (47.9-53.5)	43.4 (42.8-44.0)
30-39	62.0 (59.6-64.4)	58.7 (58.2-59.2)
40-49	66.6 (64.5-68.8)	62.9 (62.4-63.4)
50-59	70.0 (67.9-72.1)	67.6 (67.1-68.1)
60-69	68.2 (65.7-70.7)	67.0 (66.4-67.6)
70+	57.1 (54.6-59.5)	57.1 (56.5-57.7)

**Education**		

<High school	63.8 (61.5-66.1)	66.3 (65.6-67.0)
High school grad	64.1 (62.5-65.6)	62.4 (62.0-62.7)
Some college	64.0 (62.0-66.1)	59.8 (59.3-60.2)
College+	57.1 (54.7-59.6)	54.4 (54.0-54.8)

**Smoking status**		

Current	55.4 (53.4-57.3)	52.7 (52.2-53.2)
Former	68.7 (66.4-71.0)	64.2 (63.8-64.7)
Never	64.0 (62.6-65.5)	59.7 (59.4-60.0)

a All values represent percentages (95% confidence intervals). Age-adjusted to the 2002 Behavioral Risk Factor Surveillance System.

**Table 3:** Prevalence of Comorbid Conditions by Body Mass Index Category, Adults Aged ≥18, United States and Kentucky, 2000–2002 Behavioral Risk Factor Surveillance System^a^

	**Body Mass Index**				

	**Normal** **(18.5-24.9)**	**Overweight** **(25.0-29.9)**	**Obese-class 1** **(30.0-34.9)**	**Obese-class 2** **(35.0-39.9)**	**Obese-class 3** **(≥40.0)**

**Diabetes**					

Kentucky	3.7 (3.2-4.3)	7.1 (6.4-7.9)	12.1 (10.6-13.6)	19.3 (16.2-22.3)	23.7 (17.7-29.7)
United States	4.8 (4.7-5.0)	7.5 (7.3-7.7)	13.4 (13.0- 13.8)	19.8 (18.9- 20.7)	26.4 (25.0- 27.8)

**Asthma**					

Kentucky	9.9 (9.0-10.9)	10.2 (9.1-11.2)	12.8 (11.1-14.5)	15.3 (12.5-18.0)	21.8 (16.9-26.7)
United States	9.6 (9.4-9.8)	9.9 (9.7-10.1)	12.6 (12.2-13.0)	16.0 (15.2-16.8)	21.9 (20.5-23.2)

**Arthritis**					

Kentucky	28.8 (27.4-30.2)	32.9 (31.5-34.3)	40.1 (37.8-42.4)	48.6 (44.4-52.7)	57.6 (52.0-63.2)
United States	22.3 (21.9-22.6)	25.6 (25.2-26.0)	32.3 (31.8-32.9)	38.5 (37.4-39.5)	47.1 (45.4-48.7)

**High blood pressure^b^**					

Kentucky	22.9 (20.7-25.0)	32.5 (30.2-34.8)	45.2 (41.5-49.0)	49.6 (42.6-56.6)	50.1 (41.9-58.3)
United States	19.1 (18.6-19.6)	28.9 (28.3-29.4)	39.5 (38.6-40.5)	46.7 (44.9-48.4)	53.5 (51.0-55.9)

**High cholesterol^b^**					

Kentucky	24.1 (21.4-26.7)	33.6 (30.6-36.5)	34.8 (30.2-39.4)	36.5 (29.5-43.5)	27.4 (18.9-35.8)
United States	24.7 (24.1-25.3)	32.5 (31.9-33.2)	37.6 (36.5-38.7)	37.0 (35.1-39.0)	36.0 (33.4-38.7)

**Fair/poor health**					

Kentucky	20.2 (18.9-21.4)	22.4 (21.1-23.6)	29.2 (26.9-31.4)	37.7 (33.8-41.6)	47.1 (39.9-54.4)
United States	13.1 (12.8-13.3)	14.7 (14.5-15.0)	20.9 (20.4-21.4)	29.6 (28.6-30.6)	39.4 (37.9-40.9)

a All values represent percentages (95% confidence intervals). Age-adjusted to the 2002 Behavioral Risk Factor Surveillance System.

b 2001 only.

**Table 4:** Adjusted Prevalence Odds Ratios for Selected Comorbid Conditions by Body Mass Index Category, Adults Aged ⩾18, United States and Kentucky, 2000–2002 Behavioral Risk Factor Surveillance System^a^

	**Body Mass Index**				

	**Normal (referent)**	**Overweight**	**Obese-class 1**	**Obese-class 2**	**Obese-class 3**

**Diabetes**					

Kentucky	1.00	1.83 (1.49-2.26)	3.31 (2.63-4.16)	5.96 (4.51-7.89)	9.10 (6.55-12.65)
United States	1.00	1.56 (1.49-1.64)	2.93 (2.77-3.09)	4.71 (4.38-5.07)	7.13 (6.54-7.76)

**Asthma**					

Kentucky	1.00	1.10 (0.93-1.31)	1.34 (1.10-1.65)	1.61 (1.26-2.05)	2.72 (2.04-3.63)
United States	1.00	1.12 (1.09-1.17)	1.44 (1.38-1.50)	1.83 (1.72-1.94)	2.65 (2.45-2.86)

**Arthritis**					

Kentucky	1.00	1.34 (1.20-1.51)	1.88 (1.63-2.18)	2.84 (2.26-3.58)	4.26 (3.28-5.53)
United States	1.00	1.37 (1.32-1.41)	1.95 (1.87-2.03)	2.62 (2.47-2.79)	4.05 (3.73-4.39)

**High blood pressure^b^**					

Kentucky	1.00	1.74 (1.43-2.12)	3.26 (2.57-4.13)	4.18 (2.75-6.34)	6.83 (4.08-11.44)
United States	1.00	1.84 (1.76-1.93)	3.19 (3.00-3.39)	4.51 (4.12-4.94)	6.46 (5.75-7.27)

**High cholesterol^b^**					

Kentucky	1.00	1.65 (1.35-2.03)	1.73 (1.33-2.25)	2.03 (1.39-2.96)	1.60 (0.95-2.71)
United States	1.00	1.48 (1.41-1.55)	1.90 (1.78-2.01)	1.88 (1.71-2.07)	1.88 (1.67-2.13)

**Fair/poor health **					

Kentucky	1.00	1.18 (1.04-1.34)	1.74 (1.48-2.04)	2.67 (2.15-3.31)	4.59 (3.46-6.09)
United States	1.00	1.11 (1.07-1.15)	1.65 (1.58-1.72)	2.64 (2.49-2.80)	4.36 (4.04-4.72)

a All values represent odds ratios (95% confidence intervals). Adjusted for age, race, sex, education, smoking status to the 2002 Behavioral Risk Factor Surveillance System.

b 2001 only.
